# Quantification of monoterpene emission sources of a conifer species in response to experimental drought

**DOI:** 10.1093/aobpla/plx045

**Published:** 2017-08-30

**Authors:** Marvin Lüpke, Michael Leuchner, Rainer Steinbrecher, Annette Menzel

**Affiliations:** 1 Technische Universität München, Ecoclimatology, Hans-Carl-von-Carlowitz-Platz 2, 85354 Freising, Germany; 2 TUM Institute for Advanced Study, Lichtenbergstraße 2 a, 85748 Garching, Germany; 3 Karlsruhe Institute of Technology KIT, Institute of Meteorology and Climate Research, Department of Atmospheric Environmental Research (IMK-IFU), Kreuzeckbahnstraße 19, 82467 Garmisch-Partenkirchen, Germany

**Keywords:** ^13^CO_2_, *de novo* BVOC emissions, drought, dynamic plant chambers, monoterpenes, Scots pine

## Abstract

Monoterpene (MT) emissions of conifer tree species, emitted from *de novo* synthesis and storage pools, play an important role in plant ecology and physiology. During drought stress both emission sources are affected differently and with conventional measuring techniques they are difficult to separate. We investigated ^13^C labelled MT emission of eight 3-year-old Scots pine seedlings in a drought stress experiment using a dynamic gas exchange chamber system (Tree DEMON). Monoterpene, water vapour and CO_2_ gas exchange were measured for a 2-day normal watering, a 11-day treatment and a 3-day re-watering period. In each period all trees were ^13^C labelled once for 5 h. Results showed the expected decrease of MT, water vapour and CO_2_ gas exchange with decreasing soil water content. However, during re-watering water vapour and CO_2_ gas exchange recovered fast to pre-drought levels, whereas MT increased to a lower level compared to the initial non-stressed phase. The ^13^C labelling showed highly variable %^13^C values for different MTs, which ranged compound-specific from 0.5 to 95 % for unstressed trees. Overall, around 36 ± 5 % of the total emission rate originated from *de novo* synthesized MTs during the 2-day prior to stress period. During full drought, the *de novo* fraction was reduced to 3 %. For the re-watering phase *de novo* emissions recovered only partly to 20 %, while pool emissions reached pre-drought conditions. Thus, emissions of *de novo* synthesized MTs of Scots pine are down-regulated by soil drought rather than MT emissions from pools.

## Teaser

We studied with the help of 13CO2 labelling and an advanced gas exchange measurement system (Tree DEMON) the strength of de novo and pool isoprenoid emissions of Scots pine during drought and rewetting. Both emission types showed a reduction, however, the de novo emissions were more strongly affected by drought and recovered slower during rewetting. The study also showed a strongly varying ratio of pool and de novo emissions for the individual isoprenoids. Furthermore an improved emission standardization algorithm was applied to correctly standardize the emission rates from de novo and pool emissions to determine the emission potential under different drought stress states.

## Introduction

Conifer trees, which dominate colder regions of the northern hemisphere, contribute around 10 % to the total monoterpene (MT) emissions into the atmosphere (Guenther *et al.* 2012), which have a significant role in atmospheric ozone and secondary organic aerosol chemistry (Yu *et al.* 1999; Calfapietra *et al.* 2013; Emanuelsson *et al.* 2013). *Pinus sylvestris* (Scots pine) is a widely distributed conifer tree species and emits a significant amount of different MTs as well as sesquiterpenes (e.g. Janson 1992; Shao *et al.* 2001; Komenda and Koppmann 2002; Holzke *et al.* 2006; Bäck *et al.* 2012; Yassaa *et al.* 2012). The emitted MTs derive *de novo* from directly synthesized compounds as well as from prior synthesized compounds stored in pools, e.g. resin ducts in wood or leaves or in the liquid phase of the leaves (Shao *et al.* 2001; Ghirardo *et al.* 2010). Stored MT compounds also present in other pine species (e.g. Petrakis *et al.* 2001; Macchioni *et al.* 2003; Gao *et al.* 2005) protect against herbivory (Manninen *et al.* 1998; Mumm and Hilker 2006) or aerial pathogens (Gao *et al.* 2005), whereas newly synthesized compounds reduce oxidative stress (Graßmann *et al.* 2005).

Abiotic and biotic stressors (Holopainen and Gershenzon 2010; Loreto and Schnitzler 2010) can lead to both an increase and decrease of emissions. Mechanical damage can evoke emissions through, e.g., burst of resin ducts and subsequent release of compounds (Komenda and Koppmann 2002) or induced production of compounds in order to protect open wounds against infections (Fäldt *et al.* 2006). In contrast, drought reduces photosynthetic capacity and thus carbon allocation, which is then lacking for MT synthesis reducing their emission (Lüpke *et al.* 2016).

Tree seedlings are more sensitive to extended stress periods than adult trees (Niinemets 2010), since seedling carbon pools are much smaller and could be depleted during long stress phases and are then missing for the recovery. However, they have a higher plasticity than adult trees; e.g., during drought seedlings show a more anisohydric behaviour keeping stomata open longer (Mediavilla and Escudero 2004) and they morphologically adapt faster to drought stress (Royo *et al.* 2001).

Increased drought intensity and frequency during the last two decades (see, e.g., Carnicer *et al.* 2011; Spinoni *et al.* 2014) has led to severe forest diebacks often observed in *P. sylvestris* stands (Allen *et al.* 2010). In order to assess drought effects and to develop potential adaptation strategies, seedling studies have frequently been performed (see, e.g., Llusià and Peñuelas 1998; Wu *et al.* 2015; Aaltonen *et al.* 2016), since they allow easier manipulations and faster replication compared to adult trees.

Although MT synthesis only uses a small percentage of the plants’ carbon pool (Kesselmeier and Staudt 1999), its emission can be used as a proxy to measure non-invasively their stress responses (Niinemets 2010). Drought stress response can be seen in recently synthesized isoprenoids (e.g. Brilli *et al.* 2007; Wu *et al.* 2015), which mostly origin from carbon allocated by photosynthesis via the methylerythritol phosphate (MEP) pathway (Ghirardo *et al.* 2014). However, they can also be sustained via other pathways using stored carbon such as starch or sugars (Kreuzwieser *et al.* 2002; Schnitzler *et al.* 2004). These other pathways are able to contribute a substantial amount of freshly synthesized isoprenoids to the total emissions during a drought phase and they use up stored carbon as shown by Brilli *et al.* (2007) for isoprene.

Sources of MT emissions (*de novo* synthesized vs. stored in pools) are investigated by short-term ^13^C isotopic labelling of the target compounds by ^13^CO_2_ enrichment in the air surrounding the plants. ^13^C is photosynthesized and then further synthesized to MT. Since ^13^CO_2_ is less abundant in ambient air than ^12^CO_2_ (1.098 % vs. 98.892 % of total CO_2_; Dawson *et al.* 2002) and is moreover discriminated by plant photosynthesis (Farquhar *et al.* 1989), emitted compounds normally incorporate a very low amount of ^13^C. However, if pure ^13^CO_2_ is supplied for photosynthesis, this carbon isotope is enriched in the corresponding downstream compounds. Loreto *et al.* (1996), Shao *et al.* (2001) and Harley *et al.* (2014) showed that ^13^C labelling increases the compound mass by one mass unit for each labelled C-atom. This method has been used in prior studies on, e.g., broad-leaved species such as *Quercus rubra* (Delwiche and Sharkey 1993), *Quercus ilex* (Loreto *et al.* 1996; Ghirardo *et al.* 2010; Wu *et al.* 2015), *Betula pendula* (Ghirardo *et al.* 2010) and *Fagus sylvatica* (Wu *et al.* 2015). Also conifer species, known for their large MT pools, were instigated with ^13^C labelling such as *Larix decidua*. (Ghirardo *et al.* 2010), *Picea abies* (Schürmann *et al.* 1993; Ghirardo *et al.* 2010), *Pinus ponderosa* P.Lawson & C.Lawson and *Pinus nigra* (Harley *et al.* 2014), *Pinus pinea* (Noe *et al.* 2006) and *P. sylvestris* (Shao *et al.* 2001; Ghirardo *et al.* 2010; Kleist *et al.* 2012; Wu *et al.* 2015). Two analytical methods have been used to measure labelled MT emissions, as compound groups by proton-transfer-reaction mass spectrometer (PTR-MS) (e.g. Ghirardo *et al.* 2010; Harley *et al.* 2014) and by pre-concentration of labelled compounds on adsorbent tubes, allowing single MT compound detection in a GC/MS (e.g. Shao *et al.* 2001; Lüpke *et al.* 2016).

Many earlier studies investigated only ^13^C recently fixed by photosynthesis but not alternative carbon pools for isoprenoid synthesis. Ghirardo *et al.* (2010) proposed an advanced method to include both sources, which was also applied on two other pine species by Harley *et al.* (2014) and in this study.

We investigated MT emissions of Scots pine seedlings under controlled light and temperature conditions, however, with fast changes of soil water content (SWC) in a drought experiment, which comprised normal watering, a drought phase as well as a final re-watering phase.

During these three phases, three ^13^CO_2_ labelling campaigns were performed in order to test the following key hypothesis: drought stress reduces single compound MT emissions from pools and from *de novo* synthesis in a similar magnitude. We focused on the four research questions: (1) Does multi-labelling cause interferences in the %^13^C? (2) How large are the *de novo* fractions of different MT compounds? (3) How strongly do trees respond to drought stress and to stress relief by re-watering? (4) How strongly does the drought stress affect the *de novo* emissions and at which point in time emissions originates only from pools?

## Methods

### Experimental setup

The combined drought and ^13^C labelling experiment comprised two replications, from 01 July 2015 until 16 July 2015 and from 24 July 2015 until 8 August 2015, respectively, with two treated (trt) and two control (ctr) Scots pine specimen each. Measurements were performed under similar environmentally controlled conditions in a dynamic plant chamber system. The experiments were undertaken at a seasonal point when needles were fully developed and no new needle growth was observed.

#### Plant and soil material. 

Eight out of 25 morphologically similarly sized 4-year-old Scots pine trees (seed origin: Mittelfränkisches Hügelland of Germany, 49.497°N, 11.184°E) were selected for the experiment. All trees had been planted in November 2013 at an age of 2 years into 5-L pots containing a soil mixture of 70 % sand and 30 % humus. This soil mixture allowed a fast drought application. The plants were raised under greenhouse conditions and watered manually during wintertime and in summertime with a dripping water system (Netafim Ltd, Tel Aviv, Israel). The trees were fertilized with a 1.2 g L^−1^ solution (FERTY® 1, Planta Düngemittel GmbH, Regenstauf, Germany) each once per week from 15 June 2015 till 17 August 2015 in order to avoid nutrient shortage.

#### Dynamic plant chambers. 

In each replication four whole tree canopies were installed into a system with four dynamic plant chambers for gas exchange assessment called Tree DEMON (tree drought emission monitor) (see Lüpke *et al.* 2016; Lüpke *et al.* 2017). The plant chambers (~30 L volume) were made of transparent PVDF (polyvinylidene fluoride with ~97 % photosynthetically active radiation (PAR) transmissibility) mounted on a stainless steel flange. These top chambers were connected to two duraluminium ground plates, where air in- and outlets, sensors and tree stems were installed. Each chamber was supplied with 9 L_n_ min^−1^ of mass flow controlled conditioned inlet air (VOC free, humidified and constant CO_2_ mixing ratio) over stainless steel tubes with multiple micro inlets [**see Supporting Information** for detailed conditioning procedure and **Fig.** S1 photo of the Tree DEMON]. This technique generated sufficient air mixing of the chamber volume with slight overpressure to avoid air leaking in and generated steady-state conditions (see also Lüpke *et al.* 2017 for a detailed technical evaluation).

Each plant chamber was equipped with two thermocouples type K (L-0044K-IEC, Omega Engineering Ltd, Northbank, Irlam, Manchester, UK) to measure leaf temperature as well as one air temperature/relative humidity sensor (FF-IND-10V-TE1, B+B Thermo-Technik GmbH, Donaueschingen, Germany). Before the tree canopies were installed into the plant chambers, a PTFE band was wrapped around the stem (below canopy) for protection and better sealing. Trees were installed 2 days before actual sampling start in order to adjust to the plant chambers and environmental conditions.

#### Climate chamber environmental settings. 

The Tree DEMON was placed in a climate chamber which guaranteed stable environmental conditions comparable between both replications. The climate chamber was set to a constant temperature of 24 °C with a relative humidity of 50 %. Light levels were ramped to simulate a diurnal pattern with following steps starting at 0700 h local time (CEST): 1 h at 70 µmol PAR m^2^ s^−1^, 1 h at 125 µmol PAR m^2^ s^−1^, 1 h at 200 µmol PAR m^2^ s^−1^ and 8 h at 385 µmol PAR m^2^ s^−1^ and then reverse steps back to 0 µmol PAR m^2^ s^−1^, resulting in a 16-h day and 8-h night cycle. Photosynthetically active radiation was measured by one sensor (HOPL, Skye Instruments Ltd, Llandrindod Wells, Powys, UK) at mid-height of the plant chambers.

#### Watering regimes.

The drought experiment in each replication comprised the following watering regime (Table 1): the normal watering of the first 2 days encompassed 300 mL tap water manually added to each tree at 1300 h. After this phase, two of the four trees were subjected to drought from Day 3 to Day 12 by a total stop of watering, while the control trees were normally watered. At Days 13 and 14, the two treated trees were intensively re-watered in order to recover, whereas the control trees still received their normal watering. Soil water content was monitored by time domain reflectometry probes (SM300, Delta-T Devices, Cambridge, UK) installed horizontally ~5 cm above the base of the pots.

**Table 1.** Irrigation and ^13^CO_2_ labelling schemes of the experiment. *Note: Tree DEMON measurements were performed until 1300 h, whereas irrigation started after 1300 h. Two replications with each two trees per treatment and control groups were performed.

Day* (1300–1300 h)	Irrigation (mL day^−1^) of control trees (*N* = 4)	Irrigation (mL day^−1^) of treated trees (*N* = 4)	^13^CO_2_ labelling
1–3	300 (Days 1 and 2)	300 (Days 1 and 2)	Day 2
3–13	300	0	Day 8
13	300	1200	
14	300	600	
15–16	300	300	Day 15

#### ^13^C labelling.

The ^13^C labelling experiment was performed simultaneously at all trees on Days 2, 8 and 15 of each replication from 1300 to 1800 h by replacing the mass flow controlled added CO_2_ (99.995 % purity, Rießner Gase, Lichtenfels, Germany) of the supply air (~400 µmol mol^−1^ CO_2_) via a manual switch valve by ^13^CO_2_ (99.9 % ^13^CO_2_, Sigma Aldrich Chemie GmbH, Munich, Germany).

#### CO_2_ and water vapour gas exchange.

The differential gas exchange measurement of CO_2_ and water vapour was performed with an infrared gas analyser (IRGA) (CIRAS 2 DC, PP-Systems, Amesbury, MA, USA) at each chamber air in- and outlet sequentially every 5 min by a magnetic valve manifold. Since ^13^CO_2_ affected the sensitivity of the IRGA CO_2_ channel, consequently leading to biased net photosynthesis rates during the labelling experiment, CO_2_ exchange data were not analysed during the isotopic labelling experiment. Thus, steady-state gas exchange conditions in the course of the experiment were identified by data from the not affected H_2_O channel only. Transpiration and photosynthesis rates were calculated according to Caemmerer and Farquhar (1981) from the differential measurement of CO_2_ and water vapour at chamber in- and outlets by the IRGA.

#### Biomass assessment.

Biomass and leaf area of all individuals were measured after each replication. Trees were harvested and dried in an oven for 48 h at 60 °C and stem and needle dry mass were weighed separately. The specific leaf area (SLA) was determined by optical scanning of 5 g of dried needles of a tree and deriving the leaf area from the scans with ImageJ (Schneider *et al.* 2012). The whole canopy leaf area (*A*_leaf_) for each tree was determined by upscaling of SLA with the total needle mass.

### BVOC sampling and analysis

BVOC samples were taken with adsorbent tubes (ATs) for 50 min with a flow rate of 150 mL min^−1^ at 0400 and 0500 h during night and 1100 and 1200 h during day at 385 µmol PAR m^2^ s^−1^. On ^13^C labelling days, additional samples were taken at 1500, 1600, 1700 and 1800 h to monitor the labelling rate.

More precisely, outlet air of each chamber was sampled mass flow controlled (SMART 4S GCS, Vögtlin Instruments AG, Aesch, Switzerland) by a sampler string with each four ports consisting of an AT installed between two pneumatically controlled stainless steel valves (VXA2120M-01F-1-B, SMC Pneumatik GmbH, Gröbenzell, Germany). Each port separated the AT from the chamber outlet airflow until the start of the sampling period. ATs were made of inert stainless steel (Camsco, Houston, TX, USA) with a two-stage adsorbent bed containing 70 mg Tenax TA© and 40 mg Carbograph® 5TD both with a 60/80 mesh size. The sampled compounds on the AT were thermally desorbed within 12 h after sampling with a Perkin Elmer ATD 650 (Perkin Elmer, Waltham, MA, USA) by a dual-stage desorption with a cold trap [**see Supporting Information** Thermal desorption method]. Desorbed compounds were transferred over a heated glass tube to a Clarus© SQ8 GC/MS system (Perkin Elmer, Waltham, MA, USA) and separated by an Elite 5MS column (30 m length, 250 µm diameters, Perkin Elmer, USA) by a ramped temperature programme [**see Supporting Information** GC method].

The separated compounds were split into two detectors at the end of the column which both received the same amount of analyte within similar retention times. One detector was a flame ion detector (FID) running at 300 °C and the second a quadrupole mass spectrometer (MS) Clarus© SQ8 (Perkin Elmer, Waltham, MA, USA) with electro-ionization at 70 eV running in full scan mode (m/z 33 to 330). Compounds were identified by their mass spectra with the NIST library and additionally confirmed by a 16-component BVOC gas standard (C_5_–C_12_ with a mixing ratio ranging from 1.81 to 2.22 nmol mol^−1^ and expanded uncertainty ranging from 0.09 to 0.30 nmol mol^−1^, NPL, Teddington, Middlesex, UK **[see Supporting Information—Table S1]**). The gas standard was also used for calibration together with an internal standard of Δ^2^-carene (mixing ratio of 87 ± 8.7 nmol mol^−1^ expanded uncertainty, SIAD Austria GmbH, St. Pantaleon, Austria). Fifty millilitres of the internal standard were added to every AT before actual sampling in order to compensate for system fluctuations. The quantification of compounds was performed by the FID which had a detection limit ranging from 0.001 to 0.02 nmol mol^−1^. The target compounds had to be present in the gas standard and in the trees and had to be unbiased by co-eluting compounds.

#### Quantification of %^13^C.

For all MT target compounds, their ^12^C part (M12) was determined by the integrated signal area of the main fragment of the molecule obtained by the mass spectrometer, e.g., at m/z 93 for most identified MTs in this study. For the ^13^C part (M13) the sum of the integrated signal of the isotopologues from m/z 94 to m/z 100 was used, since m/z increased for each additional built-in ^13^C-atom. For some compounds other main fragments and isotopologues were used, e.g. m/z 154 for 1,8-cineole (isotopologues m/z 155 to 164) or m/z 119 for *p*-cymene (isotopologues m/z 120 to 128), respectively. The ^13^C share (%^13^C) was calculated after Equation 1:

$${\%}^{13}C=\frac{M13}{M12+M13}\;$$ (1)

Due to the presence of 1.1 % ^13^C within ambient air CO_2_, non-labelled MT molecules and their fragments contained natural occurring ^13^C. Therefore, the non-labelled %^13^C (average of 1100 and 1200 h sample) was subtracted from the most labelled %^13^C (1700 h sample) in order to obtain the pure share of the ^13^C labelling.

#### Emission rate calculation.

BVOC emission rates (nmol m^−2^ s^−1^) were calculated after Niinemets *et al.* (2011) by Equation 2:

$${\text{EM}}_{\text{sample}}\;=(\chi_{\text{out}}-\chi_{\text{in}})F_{\text{in}}\;A_{\text{Leaf}}{}^{-1}+\chi_{\text{out}\;}E$$ (2)

in which *χ*_in_ and *χ*_out_ are in- and outlet concentration (nmol mol^−1^). In the used setup *χ*_in_ is zero due to filtered inlet air. *A*_leaf_ is the leaf area (m^2^) of the tree and *F*_in_ the molar flow rate passing in the chamber (mol s^−1^) set by the inlet mass flow controller. In a further step, this emission rate is corrected by additional water vapour transpired by the plants (*E* in Equation 2 with mol m^−2^ s^−1^) which corrects the mass balance between in- and outlet air.

### *De novo* emission and pool emission standardization

*De novo* emissions rely not only on pathways using under normal conditions recently fixed carbon by photosynthesis but can also use stored carbohydrates (see, e.g., Brilli *et al.* 2007). The ^13^C labelled emission refers only to photosynthetically fixed carbon and does not incorporate *de novo* emissions from other carbon sources. However, some compounds, e.g. isoprene, 3-methyl-2-butanone (MBO) or 1,8-cineole, can be considered as purely *de novo* emitted since they have no or very small storage pools and show highly light-dependent emissions (Harley *et al* 2014; Wu *et al.* 2015). During ^13^C labelling these compounds show high ratios of fast labelled %^13^C and the remaining %^12^C is almost exclusively related to pathways with alternative carbon sources (e.g. starch). Since the *de novo* part of a mixed emission compound (pools and *de novo*) relies on similar pathways like pure *de novo* emitted compounds, a correction method used by Ghirardo *et al.* (2010) and Harley *et al.* (2014) was applied. Here, the %^13^C of compounds with mixed emissions was normalized by %^13^C of a purely *de novo* emitted compound, which was 1,8-cineole in this study. This compound is completely *de novo* emitted by Scots pine as shown by previous studies of Wu *et al.* (2015), Kleist *et al.* (2012) and Ghirardo *et al.* (2010).

Out of the derived *de novo* fraction the pool and *de novo* emission rates were calculated and standardized to 1000 µmol PAR m^−2^ s^−1^ by the following algorithms. For the *de novo* part a combined light and temperature standardization algorithm was used (Guenther 1997) as shown in Equation 3:

$${\mathrm{EM}}_{de\;novo}=\frac{{\mathrm{EM}}_{de\;novo\;sample}}{f(T_{L})*f(Q)}$$ (3)

In Equation 3, the measured *de novo* emission rate EM_*de novo* sample_ was standardized by the correction term for leaf temperature *f*(*T*_L_) to 30 °C and for the correction term *f*(*Q*) to light level of 1000 µmol PAR m^−2^ s^−1^. The used parameters for *f*(*Q*) and *f*(*T*_L_) were the same as used by Guenther (1997) [**see Supporting Information** Standardization algorithm]. The pool emission EM_pool_ was corrected by a pure temperature algorithm of Guenther *et al.* (1995) in Equation 4 with an exponential functional containing an empirical value *β* of 0.09, leaf temperature *T*_L_ in K and the standard temperature *T*_std_ of 303.15 K (30 °C):

$${\mathrm{EM}}_{\text{pool}}=\frac{{\mathrm{EM}}_{\text{pool sample}}}{e^{\left(\beta (T_{L}-T_{\text{std}})\right)}}$$ (4)

In a further step the standardized (30 °C and 1000 µmol PAR m^−2^ s^−1^) *de novo* fraction *f*_*de novo*_ was calculated by Equation 5:

$$f_{de\;novo}=\frac{{\mathrm{EM}}_{de\;novo}}{\left({\mathrm{EM}}_{de\;novo}+E_{pool}\right)}$$ (5)

During normal sampling a separation of pools and *de novo* is not possible, thus the mixed type emission was corrected by a combined algorithm (also used by, e.g., Schuh *et al.* 1997; Ghirardo *et al.* 2010; Harley *et al.* 2014) in Equation 6:

$$\mathrm{EM}=\frac{{\mathrm{EM}}_{\mathrm{sample}}}{\left(f_{de\;novo}\;f(T_{L})*f(Q))+((1-f_{de\;novo})*e^{\left(\beta (T_{L}-T_{std})\right)}\right)}$$ (6)

Since *f*_*de novo*_ highly depends on the MT synthesis capacity and should change under stress condition, a fixed constant cannot be used. In order to estimate *f*_*de novo*_ for the non-labelled emissions, a non-linear model with a Michaelis–Menten function (see Equation 7) was fitted between the standardized emission rate of 1,8-cineole and standardized *f*_*de novo*_ of each compound.

$$f_{de\;novo}=\frac{a*{\mathrm{EM}}_{1,8-\mathrm{cineole}}{}_{\;}}{b+{\mathrm{EM}}_{1,8-\mathrm{cineole}}{}_{\;}}$$ (7)

### Statistical analyses

Data processing and statistics were performed with the software R (R Core Team 2017, Version 3.3). Reported values are means of each group with the respective standard error.

The paired Wilcoxon signed-rank test was used to identify how many days after ^13^C labelling were needed to reach %^13^C of the pre-labelling period in order to identify how long the labelling signature was present (residence time). Here, the 1700 h sampling (^13^C labelling; Day 2, *N* = 8) as well as the post-labelling samples at 1200 h from Day 3 to Day 7 (*N* = 8) were compared with the 1200 h (non-labelled, Day 2, *N* = 8) sample.

The gas exchange rates of the control and stress group were compared for three phases defined by SWC as well as gas exchange of CO_2_ and water vapour of the treatment group each lasting 3 days: (I) non-stressed phase: plants were well watered and showed a stable gas exchange; (II) fully drought stressed: SWC indicated drought conditions (SWC < 0.06 m^3^ m^−3^) and gas exchange rates were close to zero and (III) recovering phase: re-watering of the treated group. For each 3-day phase two-sample Student’s *t*-tests were used to compare the physiological and environmental parameters of control and treatment groups with both replications pooled together. More specifically, averaged noon measurements of gas exchange (CO_2_, water vapour and BVOC), *T*_L_ and SWC (1100 and 1200 h) were compared (typical *N* = 12 per group).

## Results

### Morphological parameters

After harvesting, the biomass parameters measured for the control (ctr) and treatment (trt) group indicated similar sizes of the trees’ canopies. The average leaf biomass was 18.4 ± 2.0 g (ctr)/21.1 ± 2.0 g (trt) and the stem biomass 10.5 ± 2.0 g (crt)/10.6 ± 0.8 g (trt), respectively. The mean leaf area was 936.7 ± 107.2 cm^2^ (ctr)/1094.6 ± 78.2 cm^2^ (trt) and the mean height was 43.1 ± 5.2 cm/41.1 ± 1.5 cm. For both treatment groups no apparent growth of needles during the experiment was visible.

### SWC and gas exchange rates (CO_2_, water vapour, 1,8-cineole) during the experiment

**Figure 1.**
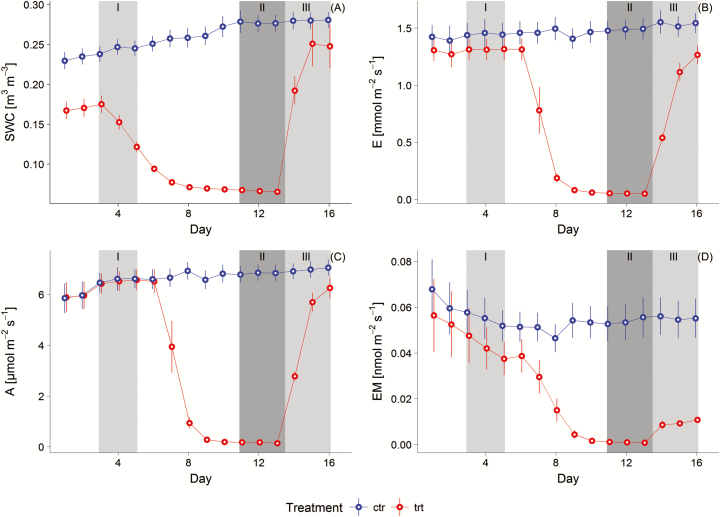
Mean daytime (1100 and 1200 h) measurements of (A) SWC, (B) net photosynthesis rate *A*, (C) transpiration rate *E* and (D) 1,8-cineole BVOC emission rate EM – light/temperature corrected by the Guenther 97 algorithm (Guenther *et al.* 1997). Mean and standard error are given for four control (ctr) and four treated (trt) trees of the two repetitions. The three periods (I to III, see also Table 1) are split into phase I non-stressed (light grey, Days 3 to 5 noon), phase II fully drought stressed (from Day 11 afternoon to Day 13 noon) and phase III re-watering (light grey area, Days 14 to 16).

Days 3 to 5 of both replications were selected for the analysis of phase I (normal watering), since at the first 2 days of replication one neon light row failed which resulted in a reduction of PAR by 50 µmol m^−2^ s^−1^ and a decrease of gas exchange (see Fig. 1C). Although all studied trees were equally watered prior to the experiment in the greenhouse, in this phase I (and also on Days 1 and 2) the trt group already showed a significant lower mean SWC between 0.18 and 0.12 m^3^ m^−3^ (Fig. 1A, see Table 2 for detailed rates and significances), likely because watering was stopped at Day 3. However, the trt group in phase I can be still considered as non-stressed, since with decreasing SWC gas exchange rates were constant till a SWC of 0.08 m^3^ m^−3^ [**see Supporting Information—**Fig. S2 for SWC gas exchange relationship]. Therefore, Days 1 to 6 can be regarded as non-stressed conditions.

**Table 2.** Mean and standard error (SE) of SWC, transpiration rate (*E*), photosynthesis rate (*A*), leaf temperature (*T*_L_) and emission rate (EM) of 1,8-cineole of both groups (trt: treatment, ctr: control) during the three phases (phase I non-stressed: Days 3 to 5, phase II fully drought stressed: Days 11 to 13 and phase III re-watering: Days 14 to 16). EM was standardized to 30 °C and 1000 µmol PAR m^−2^ s^−1^. *P* indicates the significance level of the *t*-test, while df gives the degree of freedom.

Parameter	Phase	ctr ± SE	ctr ± SE	*P*	df
SWC (m^3^ m^−3^)	I	0.24 ± 0.01	0.15 ± 0.01	<0.01	43.4
	II	0.27 ± 0.01	0.07 ± 0.02	<0.01	26.4
	III	0.28 ± 0.01	0.23 ± 0.02	<0.01	28.2
*E* (mmol m^−2^ s^−1^)	I	1.45 ± 0.06	1.31 ± 0.05	0.10	43.9
	II	1.49 ± 0.05	0.05 ± 0.00	<0.01	23.1
	III	1.54 ± 0.05	0.99 ± 0.08	<0.01	29.2
*A* (µmol m^−2^ s^−1^)	I	6.56 ± 0.22	6.50 ± 0.22	0.86	45
	II	6.82 ± 0.18	0.16 ± 0.01	<0.01	23.1
	III	6.98 ± 0.18	5.00 ± 0.37	<0.01	31.6
*T*_L_ (°C)	I	25.34 ± 0.08	25.47 ± 0.07	0.23	42.3
	II	25.52 ± 0.06	28.14 ± 0.09	<0.01	39.7
	III	25.42 ± 0.06	26.2 ± 0.16	<0.01	27.9
EM_1,8-cineole_ (nmol m^−2^ s^−1^)	I	0.055 ± 0.005	0.042 ± 0.005	0.09	44.6
	II	0.054 ± 0.004	0.001 ± 0.001	<0.01	23.1
	III	0.055 ± 0.005	0.01 ± 0.001	<0.01	24.6

In Fig. 1, an overview of the course of the experiment is given, while Table 2 shows the means of gas exchange (CO_2_, water vapour and 1,8-cineole emission) and SWC with results of the *t*-test.

In phase I, no significant differences in gas exchange between both groups were detected (see also Fig. 1B and C and Table 2 for details). In respect to BVOC emissions, only 1,8-cineole, which was completely *de novo* emitted (see Fig. 1D and Table 2), could be correctly standardized for light and temperature and is thus included in this comparison. Here, both groups showed similar 1,8-cineole emission rates.

After Days 6 and 7, gas exchanges of the trt group started to respond to decreasing SWC and declined to rates close to zero on Day 10 (see Fig. 1B and C). Consequently, Days 11 to 13 were considered as phase II with fully drought-stressed plants and SWC near the permanent wilting point. Phase II was characterized by extremely low gas exchange rates of the trt group (reduction of *A* by 98 %; *E* by 94 % and EM by 75 % from phase I to II) and by increased *T*_L_ (+2.67 °C from phase I to II), all parameters significantly differing from the ctr group (see Table 2).

Within the re-watering phase III (Days 14 to 16) SWC of the trt group increased to 0.23 ± 0.2 m^3^ m^−3^ above phase I levels. This led to an increase of gas exchange rates (see parameters *A* and *E* in Table 2), which however did not reach phase I levels. EM increased slightly, but stayed below phase I levels. Additionally, the trt group still performed significantly worse than the ctr group.

In order to assess the emission rates of compounds with pool and *de novo* fractions correctly, these had to be standardized by a mixed correction algorithm (see Methods). The algorithm parameters were obtained in the simultaneous ^13^C labelling experiment.

### ^13^C labelling of isoprenoids and ^13^C residence time

In order to check how long the ^13^C signature after the first ^13^C labelling on Day 2 was still present, the %^13^C of all trees from Day 2 (labelling day) till Day 7 (day prior to the next labelling) were investigated. Although an effect of decreasing SWC on gas exchange was observed on Day 7 (see Fig. 1), this day was included into this test in order to check if all compounds could regain pre-labelling %^13^C.

**Figure 2.**
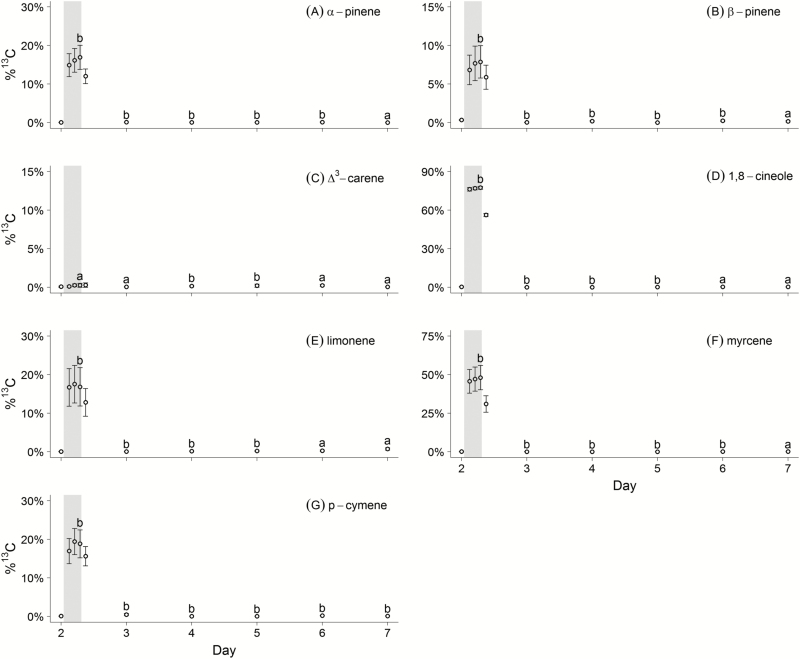
Mean %^13^C for each compound (see Methods for calculation of each compound) for (A) α-pinene, (B) β-pinene, (C) Δ^3^-carene, (D) 1,8-cineole, (E) limonene, (F) myrcene and (G) *p*-cymene of all trees before (Day 2, 1200 h), during the ^13^C labelling (grey band, Day 2, 1300 h until 1800 h) and after labelling (Days 3 to 7, 1200 h). Error bars represent the standard error. Small letters show the result of a paired Wilcoxon signed-rank test by comparing Day 2 at 1200 h (non-labelled) with the 1700 h (Day 2, labelled) and the post-labelling 1200 h samples (Days 3 to 7). Letter ‘b’ indicates significant group differences for each comparison (*P* < 0.05), while letter ‘a’ corresponds to non-significant differences, thus pre-labelling fractions.

Figure 2 shows %^13^C of seven selected compounds before, during and after the first ^13^C labelling at Day 2. All %^13^C were highest during the 1700 h sampling, yet the increase from 1600 to 1700 h sampling was small, reaching saturation. However, each compound showed significantly different %^13^C before labelling (1200 h) and in the last labelling hour (1700 h, see Fig. 2A–G, except C Δ^3^-carene). Each single compound was assigned to two groups according to their %^13^C changes: (1) compounds showing small changes, such as β-pinene (%^13^C: 7.9 ± 2.1 %), α-pinene (%^13^C: 16.9 ± 3.1 %), limonene (%^13^C: 16.9 ± 5.0 %) and *p*-cymene (%^13^C: 18.8 ± 3.6 %), (2) compounds showing strong changes, such as and myrcene (%^13^C: 48.1 ± 7.9 %) and 1,8-cineole (%^13^C: 77.4 ± 1.1 %).

Within the first 18 h after labelling, %^13^C ratios were strongly reduced and pre-labelling %^13^C levels were reached between Days 6 and 7 for most compounds (see Fig. 2; paired Wilcoxon signed-rank test). Only for *p*-cymene, the pre-labelling ratio was not reached within the 6-day time frame. In case of Δ^3^-carene, two trees showed no Δ^3^-carene emission at all, while six ones produced Δ^3^-carene. For the latter ones, labelling did not show any significant increase of %^13^C, only a slight increase on Days 4 and 5 was observed, which decreased afterwards to pre-labelling level.

### *De novo* and pool emission of isoprenoid at the ^13^C labelling days

For the three labelling days (Days 2, 8 and 15), the labelled %^13^C was calculated and normalized by the %^13^C of 1,8-cineole in order to estimate the *de novo* fraction *f*_*de novo* sample_ for all compounds. For Δ^3^-carene, which showed a very low %^13^C labelling of 0.3 %, a *f*_*de novo* sample_ of zero was used since labelled %^13^C was within the range of measurement uncertainty. Standardized pool and *de novo* emission rates (EM_pool_, EM_*de novo*_) for each compound were calculated from *f*_*de novo* sample_ for all three labelling days and treatments. EM_pool_ and EM_*de novo*_ were then used to determine the standardized *de novo* fraction *f*_*de novo*_ (see Table 3).

**Table 3.** Mean *de novo* (EM_*de novo*_, nmol m^−2^ s^−1^), pool (EM_pool_, nmol m^−2^ s^−1^), *de novo* fractions (*f*_*de novo*_) for each labelling day and treatment (ctr control, trt drought stress). SE represents the standard error. EM_*de novo*_ and EM_pool_ are corrected to standard temperature and light conditions (30 °C and 1000 µmol PAR m^−2^ s^−1^, see Equations 3 and 4). Both fractions were derived from the *f*_*de novo* sample_ which is calculated by normalizing labelled %^13^C of a specific compound by the labelled %^13^C 1.8-cineole. *f*_*de novo*_ is the fraction of EM_*de novo*_ in the total standardized emissions rate.

Compound	Treatment	Day	EM_*de novo*_ ± SE	EM_pool_ ± SE	*f*_*de novo*_ ± SE
α-Pinene	ctr	2	0.016 ± 0.003	0.026 ± 0.005	0.37 ± 0.05
		8	0.013 ± 0.001	0.022 ± 0.001	0.37 ± 0.02
		15	0.013 ± 0.002	0.023 ± 0.002	0.35 ± 0.04
	trt	2	0.016 ± 0.003	0.075 ± 0.032	0.23 ± 0.08
		8	0.006 ± 0.003	0.036 ± 0.015	0.18 ± 0.09
		15	0.005 ± 0.001	0.044 ± 0.018	0.15 ± 0.06
β-Pinene	ctr	2	0.002 ± 0.001	0.013 ± 0.005	0.09 ± 0.04
		8	0.002 ± 0	0.018 ± 0.005	0.11 ± 0.02
		15	0.002 ± 0.001	0.027 ± 0.009	0.08 ± 0.03
	trt	2	0.001 ± 0	0.024 ± 0.018	0.07 ± 0.04
		8	0 ± 0	0.007 ± 0.006	0.04 ± 0.04
		15	0 ± 0	0.013 ± 0.011	0.02 ± 0.02
Δ^3^-Carene	ctr	2	0 ± 0	0.096 ± 0.013	0 ± 0
		8	0 ± 0	0.095 ± 0.024	0 ± 0
		15	0 ± 0	0.103 ± 0.03	0 ± 0
	trt	2	0 ± 0	0.056 ± 0.049	0.01 ± 0.01
		8	0 ± 0	0.025 ± 0.022	0 ± 0
		15	0 ± 0	0.064 ± 0.06	0 ± 0
1,8-Cineole	ctr	2	0.070 ± 0.022	0 ± 0	1 ± 0
		8	0.059 ± 0.010	0 ± 0	1 ± 0
		15	0.060 ± 0.013	0 ± 0	1 ± 0
	trt	2	0.058 ± 0.024	0 ± 0	1 ± 0
		8	0.009 ± 0.005	0 ± 0	1 ± 0
		15	0.009 ± 0.002	0 ± 0	1 ± 0
Limonene	ctr	2	0.005 ± 0.002	0.006 ± 0.002	0.32 ± 0.12
		8	0.004 ± 0.001	0.006 ± 0.001	0.41 ± 0.04
		15	0.005 ± 0.001	0.008 ± 0.001	0.39 ± 0.05
	trt	2	0.004 ± 0.002	0.099 ± 0.072	0.18 ± 0.12
		8	0.001 ± 0	0.036 ± 0.027	0.17 ± 0.1
		15	0.001 ± 0.001	0.028 ± 0.017	0.10 ± 0.09
Myrcene	ctr	2	0.035 ± 0.008	0.005 ± 0	0.85 ± 0.04
		8	0.031 ± 0.005	0.006 ± 0.002	0.85 ± 0.03
		15	0.036 ± 0.008	0.008 ± 0.003	0.81 ± 0.06
	trt	2	0.033 ± 0.012	0.047 ± 0.031	0.53 ± 0.17
		8	0.011 ± 0.004	0.012 ± 0.006	0.47 ± 0.13
		15	0.010 ± 0.001	0.019 ± 0.012	0.51 ± 0.14
*p*-Cymene	ctr	2	0.005 ± 0.002	0.011 ± 0.002	0.31 ± 0.07
		8	0.005 ± 0.001	0.008 ± 0.001	0.37 ± 0.05
		15	0.005 ± 0.001	0.009 ± 0.001	0.37 ± 0.07
	trt	2	0.004 ± 0.002	0.007 ± 0.002	0.27 ± 0.11
		8	0.001 ± 0	0.003 ± 0.001	0.16 ± 0.07
		15	0.001 ± 0	0.005 ± 0.002	0.12 ± 0.05
Total	ctr	2	0.132 ± 0.037	0.158 ± 0.022	0.43 ± 0.04
		8	0.114 ± 0.019	0.154 ± 0.031	0.43 ± 0.02
		15	0.121 ± 0.026	0.178 ± 0.042	0.41 ± 0.06
	trt	2	0.116 ± 0.044	0.308 ± 0.094	0.29 ± 0.07
		8	0.027 ± 0.012	0.120 ± 0.037	0.18 ± 0.06
		15	0.026 ± 0.005	0.173 ± 0.055	0.20 ± 0.09

At all three labelling days, the total EM_*de novo*_ of the ctr group stayed constant at 0.12 ± 0.01 nmol m^−2^ s^−1^ (see Table 2) and mostly consisted of 1,8-cineole, myrcene and α-pinene. The mean total *f*_*de novo*_ of the ctr group was 0.42 ± 0.02, while the trt group showed a lower total *f*_*de novo*_ of 0.29 ± 0.07 at Day 2 (total unstressed mean *f*_*de novo*_ of 0.36 ± 0.05 of both groups pooled together), which decreased further to 0.18 ± 0.06 (Day 8) and 0.20 ± 0.09 (Day 15). The EM_*de novo*_ of the trt group was 0.12 ± 0.04 nmol m^−2^ s^−1^ at Day 2. On Day 8, EM_*de novo*_ was reduced to 0.03 ± 0.04 nmol m^−2^ s^−1^ and was on a similar level at Day 15 with 0.03 ± 0.05 nmol m^−2^ s^−1^. Furthermore, trt group %^13^C of 1,8-cineole were reduced from 94.1 ± 9.6 % (Day 2) to 71.5 ± 9.6 % (Day 8) and increased back to 84.6 ± 2.7 % (Day 15), while the ctr group showed a mean %^13^C of 96.0 ± 0.5 % over all labelling days.

On Day 2, the trt group had double total EM_pool_ (see Table 3, 0.31 ± 0.09 nmol m^−2^ s^−1^) than the crt group (0.16 ± 0.02 nmol m^−2^ s^−1^), which was mostly caused by α- and β-pinene, myrcene and limonene; however, the higher EM_pool_ of limonene was only caused by one tree. During and after the drought treatment EM_pool_ was reduced by 61.1 % to 0.12 ± 0.04 nmol m^−2^ s^−1^ on Day 8 during the drought-stressed period and by 43.9 % to 0.17 ± 0.05 nmol m^−2^ s^−1^ (Day 15 in the re-watering period), while respective values of the crt group were 0.15 ± 0.03 nmol m^−2^ s^−1^ (Day 8) and 0.18 ± 0.04 nmol m^−2^ s^−1^ (Day 15).

### Isoprenoid emission correction

**Figure 3.**
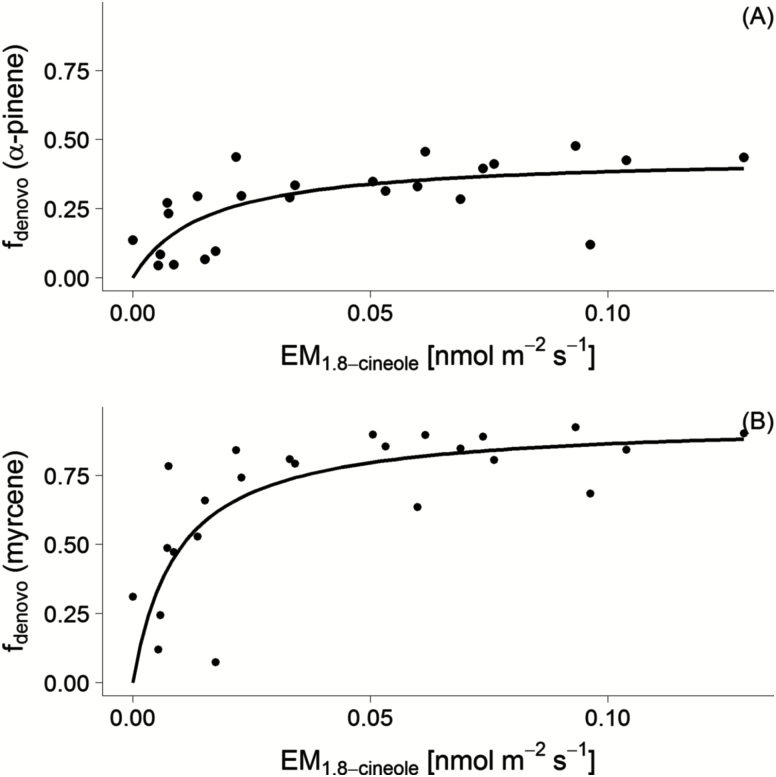
Non-linear fitting between standardized emission of 1,8-cineole and *f*_*de novo*_ of α-pinene (A) and myrcene (B). Data points were selected from the 1700 h sample at the %^13^C labelling days (Days 2, 8 and 15).

In the course of the drought experiment a decrease of the *de novo* fraction and emission was expected as indicated by the decline of the purely *de novo* emitted 1,8-cineole (see Fig. 1D) and by the decrease of *f*_*de novo*_ for many compounds during the labelling experiment at Day 8 (see Tables 2 and 3). In order to estimate *f*_*de novo*_ for the correction algorithm for mixed type emitted compounds (see Equation 5), a non-linear model based on the data of the labelling days was used (Equation 7). In Fig. 3, exemplary fits for α-pinene and myrcene are shown [**see Supporting Information—**Fig. S3 for remaining non-linear model fits].

The resulting functions were used to estimate *f*_*de novo*_ for each compound by the measured 1,8-cineole emission. Based on these *f*_*de novo*_ data, standardized emission rates could be calculated with the mixed correction algorithm. In the case of Δ^3^-carene emissions, only the pure temperature correction (*f*_*de novo*_ = 0) and in case of 1,8-cineole only the light and temperature correction (*f*_*de novo*_ = 1) were applied.

### Standardized total isoprenoid emissions during the experiment

**Figure 4.**
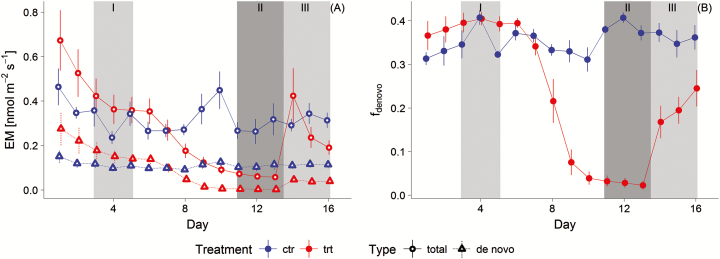
(A) Mean daytime (1100 and 1200 h) *de novo* (triangle) and total (circle) emission rates (EM) of total sum of compounds (black bar) at each day and each group (trt = treatment (*N* = 4), ctr = control (*N* = 4)) are displayed. (B) Estimated mean *f*_*de novo*_ by the non-linear model for both groups. Error bars represent the standard error.

In phase I, total BVOC emission rates (EM_total_) (see Fig. 4A) did not differ significantly between the treatment groups, but a significantly higher *f*_*de novo*_ for the trt group was revealed (see Table 4 for statistics).

**Table 4.** Mean and standard error (SE) of total emission rates (EM_total_), split into pool and *de novo* emission rates (EM_pool_, EM_*de novo*_) and the *de novo* fraction (*f*_*de novo*_) for both groups (trt: treatment, ctr: control) during the three phases (phase I non-stressed: Days 3 to 5, phase II fully drought stressed: Days 11 to 13 and phase III re-watering: Days 14 to 16). EM was standardized to 30 °C and 1000 µmol PAR m^−2^ s^−1^ by the mixed correction algorithm. *P* is showing the significance level of the *t*-test, while df gives the degree of freedom.

Parameter	Phase	ctr ± SE	trt ± SE	*P*	df
EM_total_ (nmol m^−2^ s^−1^)	I	0.32 ± 0.01	0.38 ± 0.01	0.20	44.4
	II	0.28 ± 0.01	0.06 ± 0.02	<0.01	26.3
	III	0.32 ± 0.01	0.28 ± 0.02	0.45	31.0
EM_pool_ (nmol m^−2^ s^−1^)	I	0.21 ± 0.03	0.23 ± 0.02	0.58	43.0
	II	0.18 ± 0.02	0.06 ± 0.01	<0.01	29.1
	III	0.20 ± 0.02	0.24 ± 0.05	0.48	27.1
EM_*de novo*_ (nmol m^−2^ s^−1^)	I	0.11 ± 0.01	0.16 ± 0.2	0.03	34.5
	II	0.11 ± 0.01	0 ± 0	<0.01	23.2
	III	0.11 ± 0.01	0.04 ± 0	<0.01	35.7
*f*_*de novo*_	I	0.36 ± 0.01	0.40 ± 0.01	0.02	40.6
	II	0.39 ± 0.01	0.03 ± 0.01	<0.01	44.0
	III	0.36 ± 0.02	0.2 ± 0.2	<0.01	39.9

Increasing water deficit in phase II decreased EM_total_ and EM_pool_ of the trt group significantly and EM_*de novo*_ to almost zero compared to the ctr group emitting constant EM_*de novo*_ and only marginally decreased EM_pool_.

In phase III, EM_total_ of the trt group increased after recovering and did not differ significantly from the ctr group. In detail, however, *f*_*de novo*_ and thus EM_*de novo*_ were significantly lower than the control, while EM_pool_ was similar. The treatment *f*_*de novo*_ recovered only to 50 % of the phase I fraction. On Day 14, drought-stressed trees showed a burst of EM_total_ of 0.42 nmol m^−2^ s^−1^ compared 0.24 and 0.19 nmol m^−2^ s^−1^ at the following Days 15 and 16, which was mainly related to increased EM of Δ^3^-carene, α-pinene, *p*-cymene, myrcene and limonene coming from the pool. The split-up of the emissions into the *de novo* and pool parts clearly revealed that initially high EM as well as some peaks afterwards were due to EM_pool_ (see Fig. 4).

## Discussion

### Does multi-labelling cause interferences in the %^13^C?

Overall, the effect by stored ^13^C was negligible after 18 h and vanished completely after 5 to 6 days for most compounds except *p*-cymene and showed that multiple short interval labelling is feasible without causing interferences in the %^13^C by the prior ^13^C labelling.

In detail, the temporal behaviour in first 18 h complied with the results of Shao *et al.* (2001). Some of the short-term effects should however be discussed further. Shortly after the labelling stopped, the increased %^13^C in the target compounds might be explained by lag effects of the system as well as by labelled precursor substances, e.g. geranyl diphosphate, as shown for Scots pine by Ghirardo *et al.* (2010). Another possibility is that some of the compounds synthesized during ^13^C labelling were stored in non-specified short-term pools (Noe *et al.* 2006). The slow return to pre-labelling %^13^C levels over 5 to 6 days may be due to residual ^13^C fixed in primary metabolites which are then used in the alternative MT synthesis pathways (see Brilli *et al.* 2007). This reuse of ^13^C labelled carbohydrates explained the 2-day delayed increase of %^13^C for Δ^3^-carene, which is obviously a compound not related to current photosynthesis.

### How large are the *de novo* fractions of different MT compounds?

The multiple ^13^C labelling showed that the fraction of the *de novo* emission can range between 0 and 1, depending on the specific compound. This high variability of *f*_*de novo*_/%^13^C between single compound species was also reported for other pine and conifer species by Shao *et al.* (2001), Kleist *et al.* (2012), Harley *et al.* (2014) and Wu *et al.* (2015).

The determined %^13^C agreed with studies of Shao *et al.* (2001), Ghirardo *et al.* (2010) and Kleist *et al.* (2012) in case of α-pinene and for the almost non-labelled Δ^3^-carene. However, the total *f*_*de novo*_ was lower in our study (42 % for the control/29 % for the treatment) compared to 58 % reported by Ghirardo *et al.* (2010). This can be well explained by different shares of *de novo*/pool emitting plant tissue (e.g. whole tree canopy including stem vs. branch cuvettes on twigs). Further, the different plant parts can differ in their composition (Ghirardo *et al.* 2010), size of storage pools (Manninen *et al.* 2002) and their capability for synthesis of new compounds due to access to free carbon/precursor pools. Many of the lower labelled compounds, such as α-pinene and β-pinene, are known to reside in large amounts in storage pools such as needle resin ducts (see, e.g., Hiltunen and Laakso 1995; Manninen *et al.* 2002; Achotegui-Castells *et al.* 2013). Thus, the lower %^13^C can be explained by the high pool fraction interfering with the labelled signature.

Besides, there are also methodological differences with unknown effects on total *f*_*de novo*_: our study had to discard compounds with a low abundance as well as co-eluting compounds, whereas Ghirardo *et al.* (2010) used a PTR-MS, measuring the sum of MTs and thus can report the total *de novo* fraction.

The reason for certain compounds to predominantly originate from pools might be linked to permanently required functions (see Langenheim 1994; Cheng *et al.* 2007), whereas *de novo* dominated compounds are only required to combat shortly induced stress (Loreto *et al.* 2010; Niinemets 2010).

### How strongly do trees respond to drought stress and to stress relief by re-watering?

The drought application resulted in a decline of *A*, *E* and the purely *de novo* emitted 1,8-cineole, but also of EM_total_. This drought-induced reduction of MT emission of Scots pine was equally observed by Wu *et al.* (2015) for 1,8-cineole and by Lüpke *et al.* (2016) for EM_total_. This response was also confirmed for other tree species (e.g. Llusià, and Peñuelas 1998; Llusià *et al.* 2011; Šimpraga *et al.* 2011; Lüpke *et al.* 2017). The short period of re-watering led only to a partial recovery of *A* and *E* compared to a preceding experiment on the same species (Lüpke *et al.* 2016) with similar environmental settings, gas exchange rates during stress and re-watering were lower, but within the same magnitude. This difference might be related to different soil material used (complete organic vs. predominantly sandy material in this study), plant age, investigated months (end of August and October vs. July) and different provenances studied (Italy, Spain and North-Eastern Germany vs. Southern Germany).

The MT 1,8-cineole which is almost exclusively synthesised *de novo* (~77.4 labelled %^13^C) (see also of Ghirardo *et al.* 2010; Kleist *et al.* 2012; Wu *et al.* 2015) revealed to be a good indicator compound for the drought impacts via reduced gas exchange. During the ^13^C labelling on Day 8, when several treated plants responded already to shortages in SWC by, e.g., a drop in photosynthesis to 1/6, 1,8-cineole showed a reduction of %^13^C to 71.5 % and drop in EM to 1/3. In the fully drought-stressed phase, EM of 1,8-cineole was almost zero. Brilli *et al.* (2007) showed a similar reduction for isoprene emission on drought-stressed poplar trees despite a much reduced photosynthesis and related the ongoing isoprene emission to using stored carbon. An effect of stomatal conductance on EM of MTs is not very likely, since partial pressures of the internal leaf gaseous space increase with stomata closure, and consequently the EM flux is sustained (Niinemets and Reichstein 2003; Harley *et al.* 2014).

During re-watering, EM of 1,8-cineole showed a lagged and moderated response with only reaching 25 % of the initial rates, whereas photosynthesis had already recovered to 77 % showing that re-available carbon from photosynthesis is not used immediately in 1,8-cineol synthesis. Preferably, recently allocated carbon might then be used for other processes than MT synthesis such as maintenance, growth (Wiley and Helliker 2012) or refilling of carbon pools (Brilli *et al.* 2007).

Quite interesting is the pronounced spike in pool emission on Day 14 after re-watering, a phenomenon which has been described by Brilli *et al.* (2007) for isoprene. Here, the increased EM during re-watering was related to still active alternative pathways/enzymes which based on stored and newly synthesised carbon allowing the quick production and release of terpenoids during the first re-watering phase. However, such a emission burst might result from a rapid change of the MT partial pressure until a new equilibrium between outside air and leaf gas space was reached after the initial stomata opening after re-watering (Niinemets and Reichstein 2003). Further, due to increase of the volume of water leading organs during re-watering the oleoresin pressure in woody plant parts is increased shortly (Rissanen *et al.* 2016) and thus increasing emissions from stored MTs.

### How strongly does the drought stress affect the *de novo* emissions and at which point in time emissions originates only from pools?

In case of EM_total_, drought stress reduced both pool and *de novo* emission evident on labelling Day 8 and during full drought within phase II, with much stronger effects on the *de novo* part. The major reduction of the *de novo* emission is tightly linked to the reduction of recently synthesized carbon by photosynthesis (Brilli *et al.* 2007), while the still ongoing *de novo* emission is sustained by carbon from other sources through alternative pathways (Kreuzwieser *et al.* 2002; Schnitzler *et al.* 2004; Ghirardo *et al.* 2014).

The reduction of pool emissions by drought might be caused by a *de novo* part which could not be assessed by ^13^C-pulse-labelling and was thus included in the pool part and/or by a decreased xylem water potential in the woody parts affecting EM as shown by Rissanen *et al.* (2016) and Vanhatalo *et al.* (2015) for stem emissions. A similar effect was revealed in our study for the pure pool emitted Δ^3^-carene, which increased during the re-watering phase probably caused by refilling the xylem.

### Standardization of emission rates

In order to compare MT emissions from the control and drought-impacted groups, a mixed emission correction algorithm was applied with a non-linear model to predict *f*_*de novo*_ for each individual compound. A fixed correction on the EM_total_ was rated as not suitable for Scots pine showing chemo species with different compound compositions (see, e.g., Bäck *et al.* 2012; Lüpke *et al.* 2016). In its current implementation the applied correction method is a quite coarse approach, which could be improved using more measurements especially in the low *f*_*de novo*_ range during drought. Additionally, also other pure *de novo* compounds, such as isoprene or MBO as suggested by Ghirardo *et al.* (2010) or Harley *et al.* (2014), should be considered for this approach, especially in conditions where 1,8-cineole emissions are very low.

### Caveats in the experiment

Some caveats should be discussed, although the effect on the results is likely to be minor. The Tree DEMON was designed for 5 L pots, likely reducing plant growth in long-term experiments (see, e.g., Passioura 2006; Poorter *et al.* 2012), but in case of this study it was advantageous by achieving drought conditions faster. The varying plant water uptake led to some SWC variations. However, this issue was eased by the analysis of the relation between SWC and gas exchange of CO_2_ revealing a constant gas exchange of Scots pine until a SWC threshold of 0.08 m^3^ m^−3^. As reported in other studies (Loreto *et al.* 2000; Komenda and Koppmann 2002) mechanical plant damage, which is difficult to avoid even with careful handling, enhanced pool emissions in the beginning. Small variations in temperature caused by differences in transpiration did not affect the results due to temperature correction of the emission rate. Thus, the technical limitations were solved sufficiently and should not have affected any of the results.

The largest caveat is linked to the inherent variability within the specimen of the studied provenance. Four replicates in each treatment group comprising even two chemo species may have influenced the results of single compound emissions. To account for this observation, the drought effect was discussed on the total *de novo* and pool emissions and the purely *de novo* emitted 1,8-cineole.

### Future research

In this study, chemo species led to a high variability for specific single compound emissions. Since it is so far unknown if chemo species have different *de novo* shares, future studies should first screen a larger set of trees and split drought stress and ^13^C labelling experiments by chemo species.

In future studies, the tipping point between moderate stress and full drought response should be higher temporally resolved to improve modelling of such events. Further, to observe full emissions recovery a longer recovery period is needed in future studies.

A so far less and difficult to investigate issue is how adult tree and young tree emissions respond to similar applied drought, since both have different access to stored carbon and water and show a different plant growth dynamic. This would require a field site with both age types available and the possibility to investigate both age groups under similar conditions, a technical challenge and huge effort.

## Conclusions

In our study, for the first time, information from ^13^C labelling was used to improve the standardization of emission rates of single compound emissions with varying *de novo* synthesis. This method could provide a methodological improvement also for other mixed type emitting tree species; however, due to the large variation of emitted compounds and their *de novo* fractions individual measurements are needed.

The experiment revealed that both pool and *de novo* emissions were affected by drought stress; however, *f*_*de novo*_ highly varied with compound and physiological state. Since ^13^C labelling is expensive and complex, it was compared with the night-day difference method. This method was more prone to disturbances and should be applied only if environmental control can be regulated very precisely. Drought stress reduced MT emission from *de novo* synthesis more than from pools; however, the emission of single compounds with higher *de novo* fractions as such and with protective functions against drought was preferentially supported. A more detailed investigation on different plant parts, optimally simultaneously, during drought stress application, will allow a better understanding of the different MT pools and responses to drought.

## Supporting Information

The following additional information is available in the online version of this article—

**Figure S1.** Photo of the Tree DEMON.

**Figure S2.** Relationship between soil water content and gas exchange.

**Figure S3.** Non-linear model fits for single compound *f*_*de novo*_ and 1,8-cineol and fitting parameters.

**Table S1.** Mixing ratios of single compound of the calibration gas standard and target compound.

## Sources of Funding

This study was financed by the European Research Council under the European Union Seventh Framework Program (FP7/2007-2013/ERC grant agreement No. 282250). This work was supported by the German Research Foundation (DFG) and the Technical University of Munich (TUM) in the framework of the Open Access Publishing Program.

## Contributions by the Authors

M.L. planned and ran the experiment and performed the data analysis. M.L. was the main author of this manuscript. R.S., M.L. and A.M. conceived the study, participated in its design and coordination and helped to draft the manuscript. All authors read and approved the final manuscript.

## Conflicts of Interest

None declared.

## Supplementary Material

Supporting Information
