# Supplementary material for: Quantification of monoterpene emission sources of a conifer species in response to experimental drought
Source: AoB Plants. 2017 Aug 30;9(5):plx045. doi: 10.1093/aobpla/plx045 (PMC5632518; doi:10.1093/aobpla/plx045)
Supplement: Supporting Information [file plx045_suppl_Supporting_Information.docx]

**SUPPORTING INFORMATION**

**Methods**

**Thermal desorption method:**

A Perkin Elmer ATD 650 (Perkin Elmer, USA) was used to thermally desorb the sampled compounds from the adsorption tube (AT) with a dual stage desorption. The inert stainless steel AT (Camsco, Huston, Texas, USA) consisted of a two-stage adsorbent bed containing 70 mg Tenax TA© and 40 mg Carbograph®5TD both with a 60/80 mesh size and were preconditioned at 310°C for around 20 min each with a helium purge flow 60 ml min^-1^ after each thermal desorption within the TD. Before desorption the sample AT were dry purged for 5 min with 50 ml_n_ min^-1^ helium (99.999% purity, Westfalen AG, Münster, Germany) which was previously purified to 99.9995% purity by an additional multi-stage gas purifier (SGE Analytical Science, Australia). Dry purging reduced potential humidity and oxygen left in the AT. The AT were subsequently desorbed for 15 min at 280°C with 25 ml_n_ min^-1^ and a 5 ml_n_ min^-1^ inlet split onto a -30°C Peltier cooled trap (Air Monitoring Trap©, Perkin Elmer, Massachusetts, USA) where the extracted compounds were prefocused. Finally, compounds focused on the cold trap were desorbed by ballistic heating (40°C s^-1^) to 300°C (and 10 min hold) with an outlet split of 2 ml_n_ min^-1^ and a flowrate of 1.5 ml_n_ min^-1^ onto a heated glass lined transfer line (255°C) to the GC inlet (see next section).

**GC method:**

The following GC temperature program was applied: 4 min hold at 40°C, with the first ramp to 100 °C with 15°C min^-1^, followed by a second ramp to 160°C with 5°C min^-1^ hold for 0 min and a final ramp to 240°C with 20°C min^-1^ with a hold time of 4 min at the end.

**Air supply conditioning:**

Supply air was conditioned by the following steps: (a) pressurized air from an air compressor was cleaned from background VOCs by a zero air generator (PEUS instruments GmbH, Gaggenau, Germany) and activated carbon (VWR International GmbH, Ismaning, Germany), (b) pressure was stabilized to a constant three bar and VOC free air was humidified with ultrapure water and then scrubbed of CO_2_ with soda lime, (c) operation concentration of 400 µmol mol^-1^ of CO_2_ was achieved by adding CO_2_ (99.995 % purity, Rießner Gase GmbH, Lichtenfels, Germany) over a mass flow controller (SMART6 GSC, Vögtlin Instruments AG, Aesch, Switzerland) into the system air. Via a switch valve, ^13^CO_2_ (99.9 % ^13^CO_2_, Sigma Aldrich Chemie GmbH, Taufkirchen, Germany) could be added to perform the isotopic labelling.The preconditioned air was fed mass flow controlled with 9 l_n_ min^-1^ into each plant camber (SMART4S GSC, Vögtlin Instruments AG, Aesch, Switzerland).


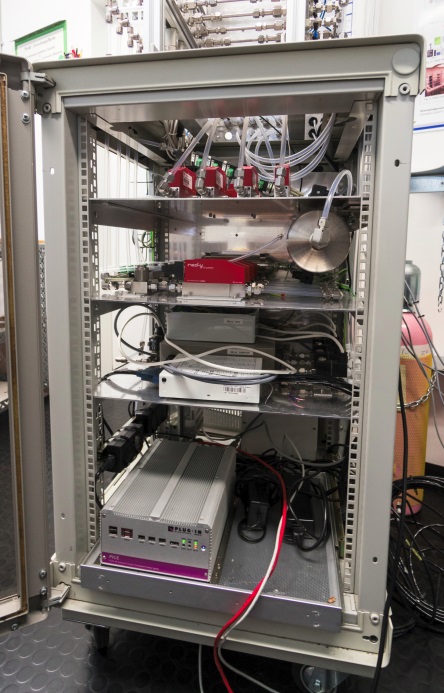


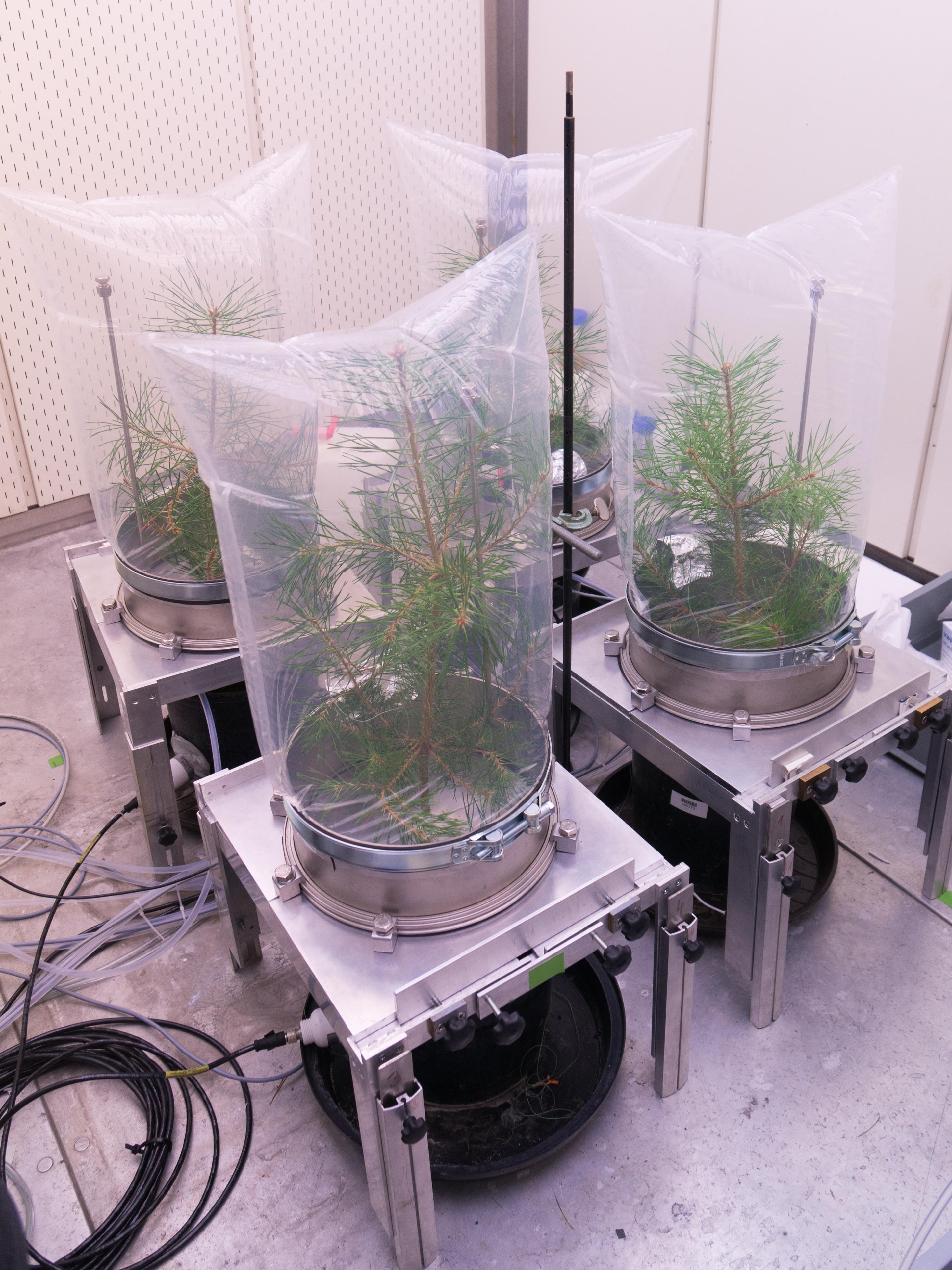


**Figure S1:** Exemplary photo of the Tree DEMON plant chambers (right) and the Tree DEMON system (left) containing samplers, controlling and air distribution. Air conditioning is not shown.

**Table S1**.Mole fractions of the NPL gas standard with expanded uncertainties

| **Substance** | Mole fraction ± uncertainty (k =2) (nmol mol^-1^) | Target compound | **Substance** | Mole fraction ± uncertainty (k =2) (nmol mol^-1^) | Target compound |
| --- | --- | --- | --- | --- | --- |
| **benzene** | 2.00 ± 0.10 | - | **myrcene** | 2.22 ± 0.11 | X |
| **toluene** | 1.98 ± 0.10 | - | **cis-ocimene** | 2.02 ± 0.30 | - |
| **m-xylene** | 2.09 ± 0.10 | - | **p-cymene** | 1.92 ± 0.10 | X |
| **p-xylene** | 2.13 ± 0.11 | - | **1,8-cineole** | 1.81 ± 0.09 | X |
| **o-xylene** | 2.15 ± 0.11 | - | **limonene** | 2.00 ± 0.10 | X |
| **isorprene** | 1.98 ± 0.10 | X | **Δ^3^-carene** | 2.09 ± 0.10 | X |
| **α-pinene** | 2.01 ± 0.11 | X | **camphor** | 2.00 | - |
| **β-pinene** | 1.90 ± 0.09 | X |  |  |  |

**Standardization algorithm**In order to standardize the emission rate to PAR intensity of 1000 µmol m^-2^ s^-1^ and temperature of 30°C the algorithm in equation S1 was used (see Guenther et al. 1997 for more detailed description)

Equation S1: ${EM}_{std} =\frac{EM}{f_{Tl} f_{Q}}$

Standardization algorithm for leaf temperature f(T_L_) and light f(Q)

Equation S2: $f_{\left( Tl \right)}=\frac{exp\left[ \frac{C_{T_{1}}(T_{L}-T_{S})}{RT_{S}T_{L}} \right]}{C_{T3}+exp\left[ \frac{C_{T_{2}}(T_{L}-T_{M})}{RT_{S}T_{L}} \right]}$

With following parameters used:
Constants of activation and deactivation energy:
 C_T1_ = 95000 J mol^-1^ C_T2_ = 230000 J mol^-1^
 C_T3_ = 0.961 J mol^-1^Gas constant: R = 8.314 J mol^-1^ K^-1^Optimum temperature: T_m_= 314 K
Standard temperature: T_S_= 303.15 K
Light dependent emission correction algorithm

Equation S3: $f\left( Q \right)=\frac{C_{L1}\alpha Q}{\sqrt{1+\alpha^{2}Q^{2}}}$

With scaling parameter C_L1_ = 1.066 and quantum yield α= 0.0027

**Results**


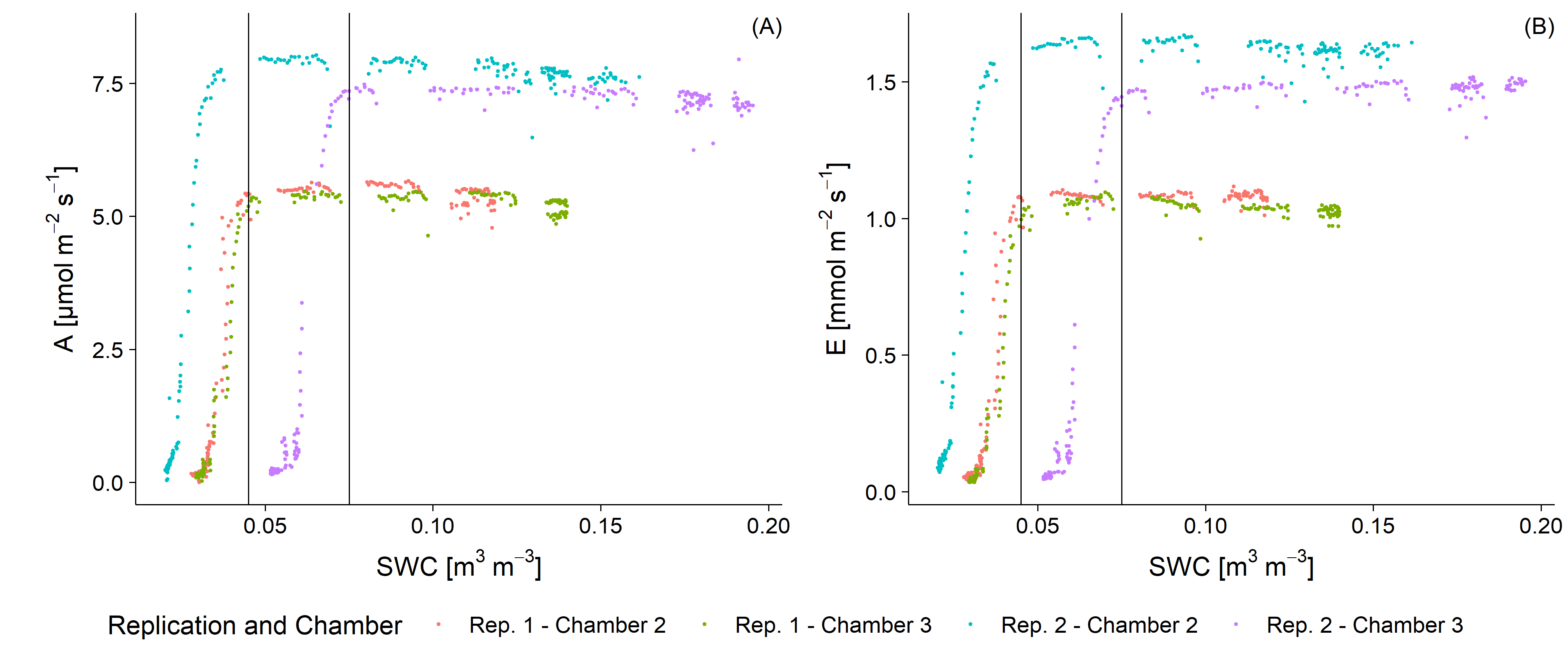


**Figure S2:** Soil water content (SWC) and photosynthesis rate (A) (Plot A) / transpiration rate (E) (Plot B) relationship. Average 5 min gas exchange rates (gas exchange was measured 5 min sequentially at each chamber). Data was selected from day 1 to 12 with PAR above 350 µmol m^-2^ s^-1^ to exclude the lamp failure. Only chambers with drought stress application are shown, split into chamber number and replication. Vertical lines represent threshold on which a gas exchange rate was observed.


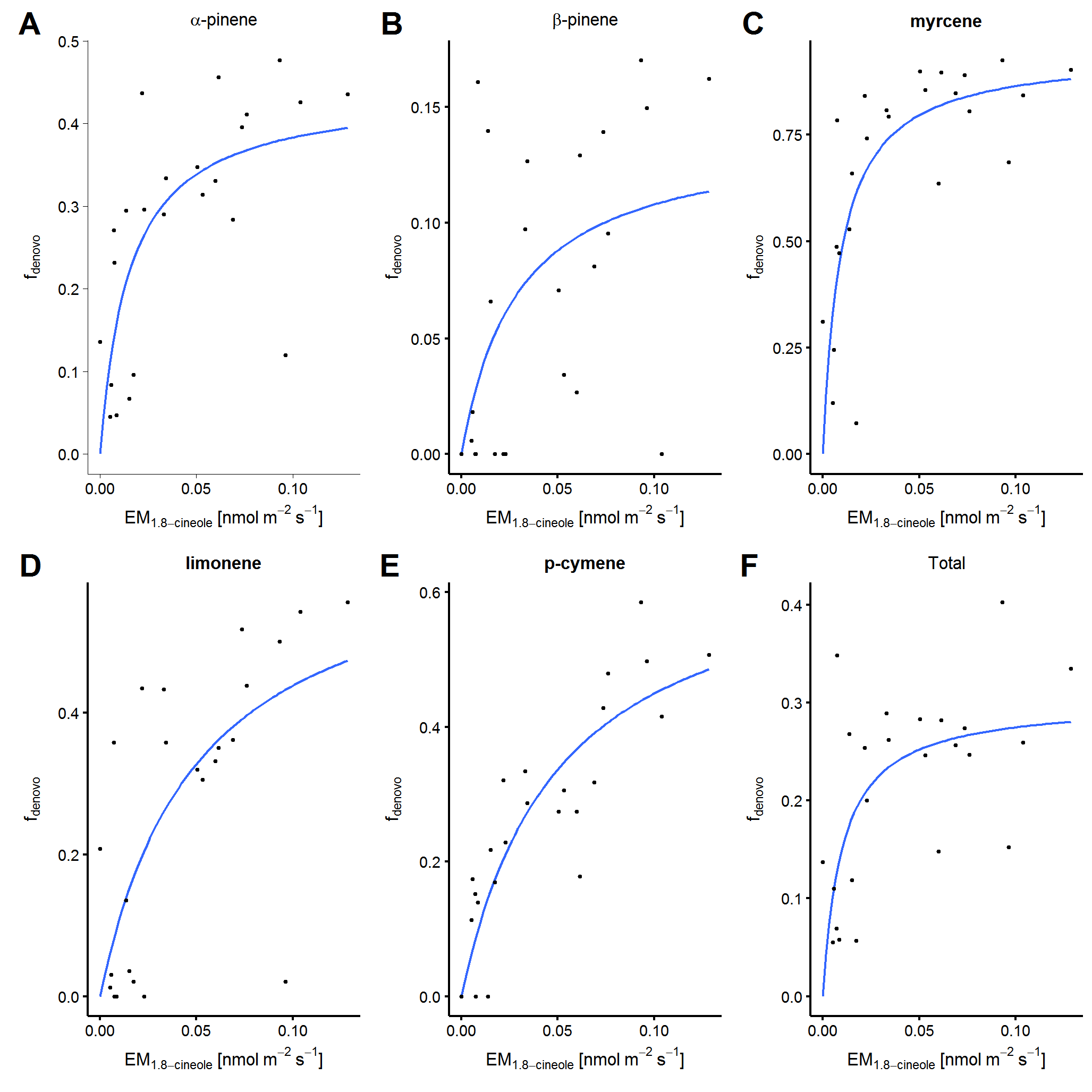


**Figure S3:** Non-linear fitting between standardized emission of 1,8-cineole for and f*_denovo_* of each target compound (A to E) and total (F). Data points were selected from the 17:00 h sample at the %^13^C labelling days.
